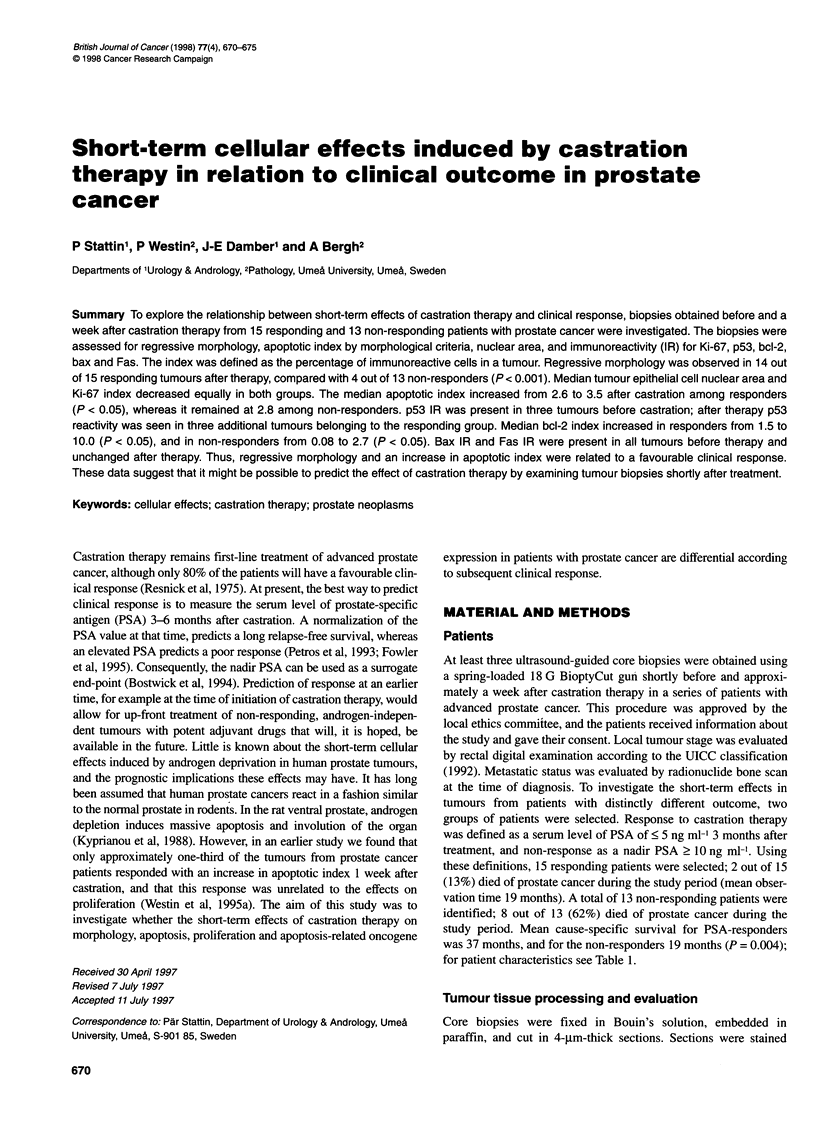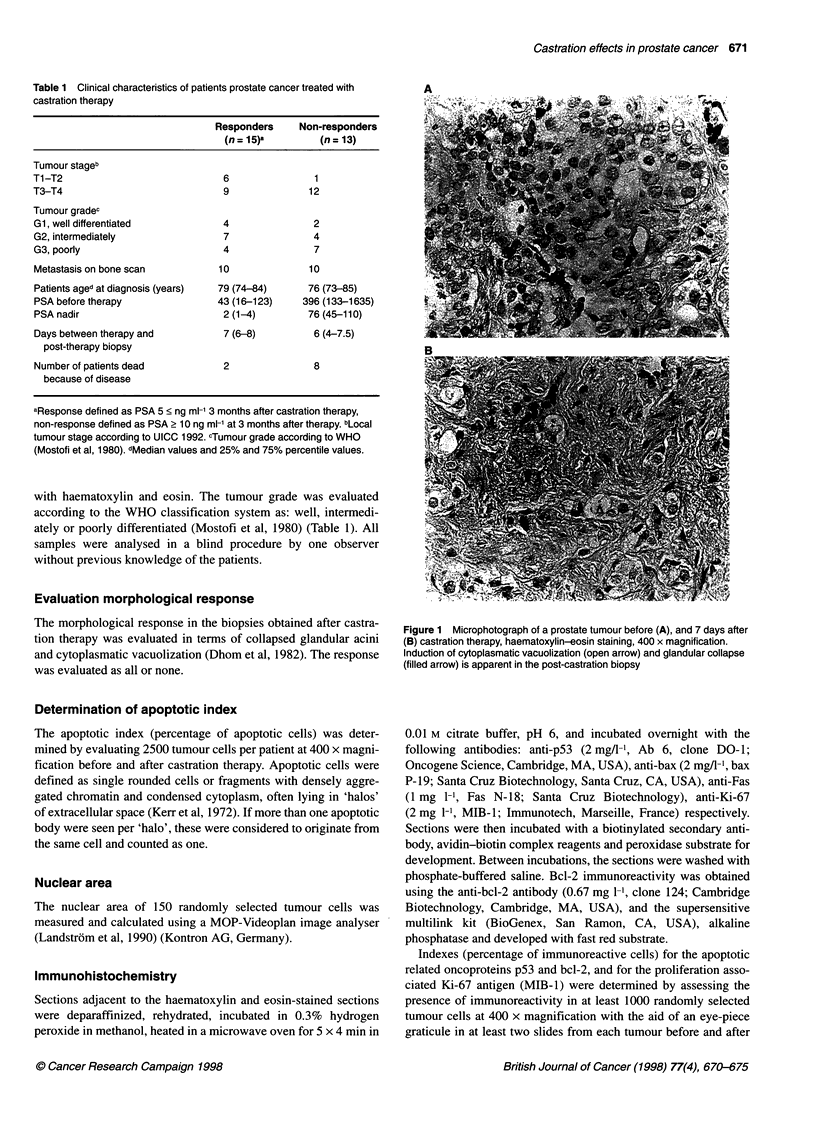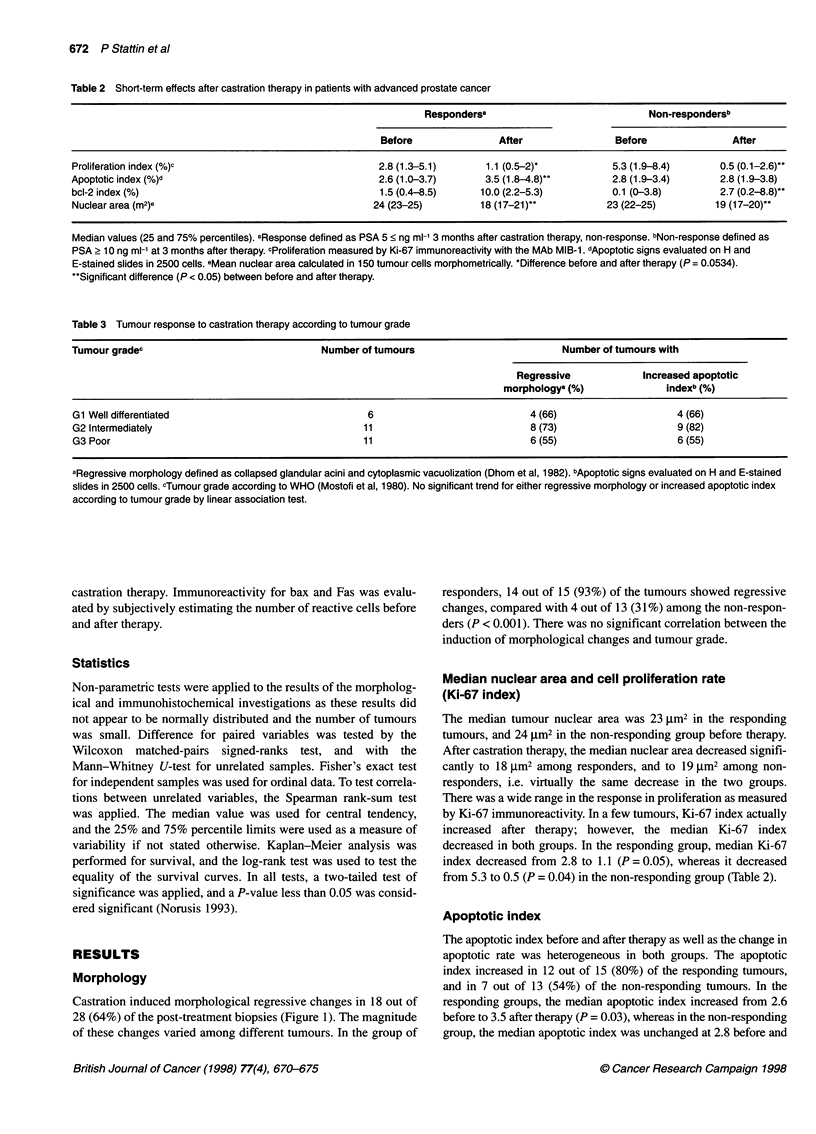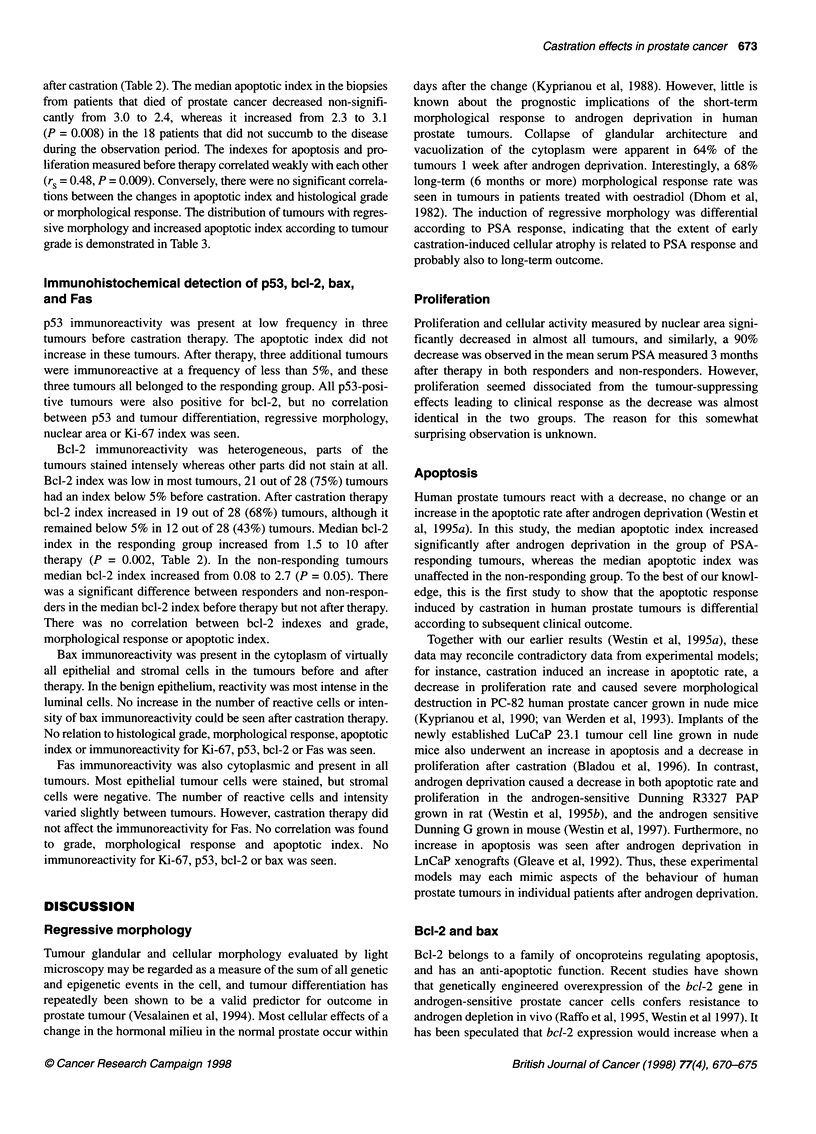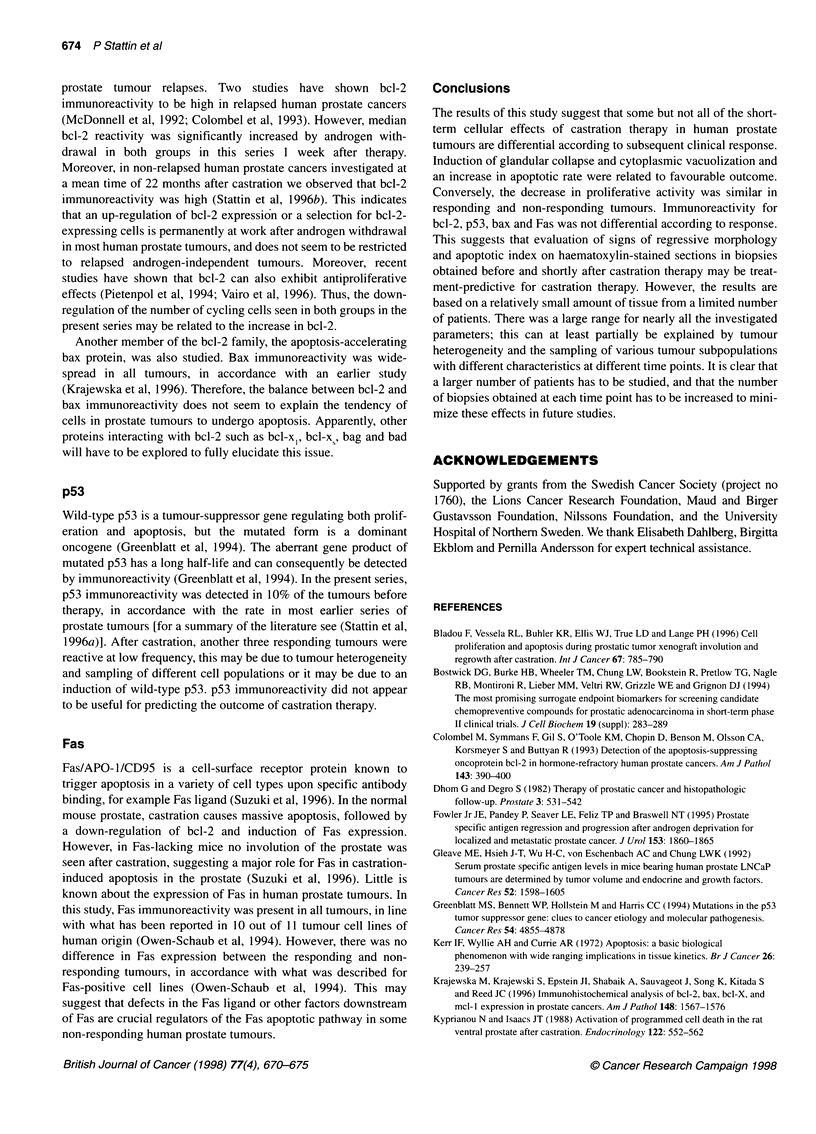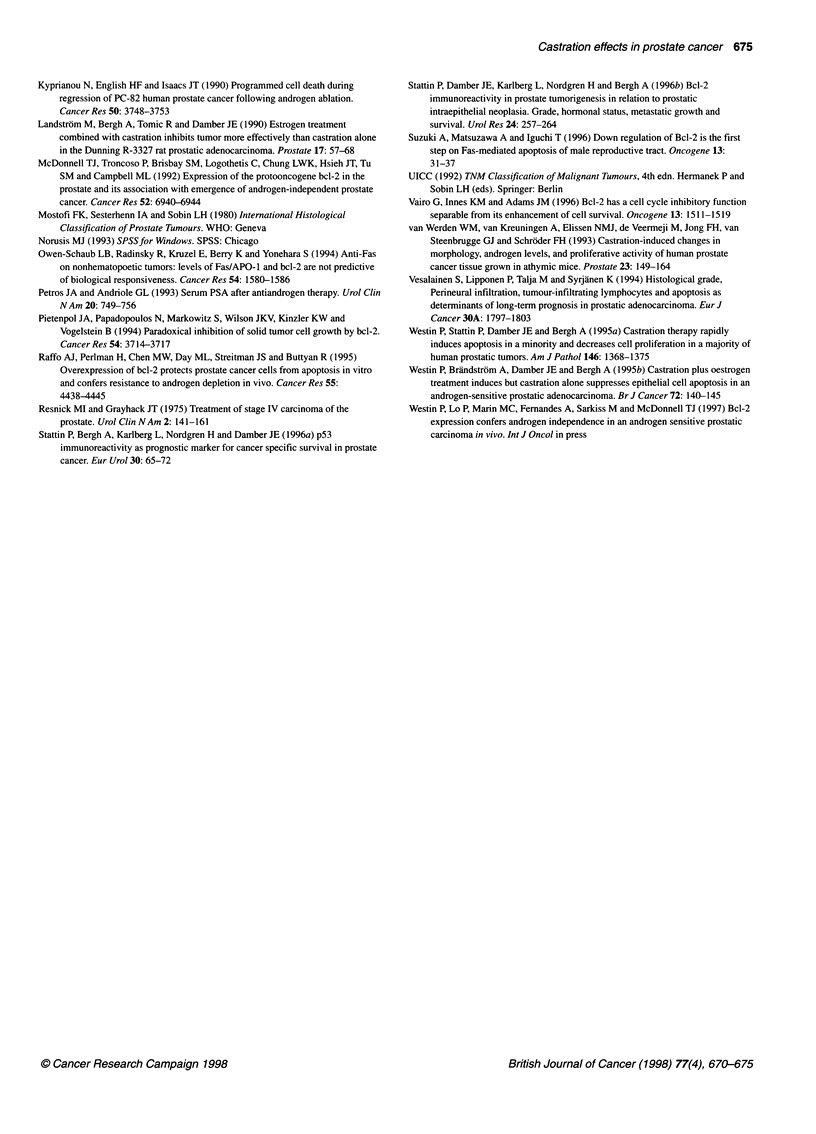# Short-term cellular effects induced by castration therapy in relation to clinical outcome in prostate cancer.

**DOI:** 10.1038/bjc.1998.107

**Published:** 1998-02

**Authors:** P. Stattin, P. Westin, J. E. Damber, A. Bergh

**Affiliations:** Department of Urology & Andrology, UmeÃ¥ University, Sweden.

## Abstract

**Images:**


British Journal of Cancer (1998) 77(4), 670–675
© 1998 Cancer Research Campaign

### Short-term cellular effects induced by castration therapy in relation to clinical outcome in prostate cancer

**P Stattin[1], P Westin[2], J-E Damber[1] and A Bergh[2]**

Departments of [1]Urology & Andrology, [2]Pathology, Umeå University, Umeå, Sweden

**Summary** To explore the relationship between short-term effects of castration therapy and clinical response, biopsies obtained before and a week after castration therapy from 15 responding and 13 non-responding patients with prostate cancer were investigated. The biopsies were assessed for regressive morphology, apoptotic index by morphological criteria, nuclear area, and immunoreactivity (IR) for Ki-67, p53, bcl-2, bax and Fas. The index was defined as the percentage of immunoreactive cells in a tumour. Regressive morphology was observed in 14 out of 15 responding tumours after therapy, compared with 4 out of 13 non-responders ($P < 0.001$). Median tumour epithelial cell nuclear area and Ki-67 index decreased equally in both groups. The median apoptotic index increased from 2.6 to 3.5 after castration among responders ($P < 0.05$), whereas it remained at 2.8 among non-responders. p53 IR was present in three tumours before castration; after therapy p53 reactivity was seen in three additional tumours belonging to the responding group. Median bcl-2 index increased in responders from 1.5 to 10.0 ($P < 0.05$), and in non-responders from 0.08 to 2.7 ($P < 0.05$). Bax IR and Fas IR were present in all tumours before therapy and unchanged after therapy. Thus, regressive morphology and an increase in apoptotic index were related to a favourable clinical response. These data suggest that it might be possible to predict the effect of castration therapy by examining tumour biopsies shortly after treatment.

**Keywords:** cellular effects; castration therapy; prostate neoplasms

Castration therapy remains first-line treatment of advanced prostate cancer, although only 80% of the patients will have a favourable clinical response (Resnick et al, 1975). At present, the best way to predict clinical response is to measure the serum level of prostate-specific antigen (PSA) 3–6 months after castration. A normalization of the PSA value at that time, predicts a long relapse-free survival, whereas an elevated PSA predicts a poor response (Petros et al, 1993; Fowler et al, 1995). Consequently, the nadir PSA can be used as a surrogate end-point (Bostwick et al, 1994). Prediction of response at an earlier time, for example at the time of initiation of castration therapy, would allow for up-front treatment of non-responding, androgen-independent tumours with potent adjuvant drugs that will, it is hoped, be available in the future. Little is known about the short-term cellular effects induced by androgen deprivation in human prostate tumours, and the prognostic implications these effects may have. It has long been assumed that human prostate cancers react in a fashion similar to the normal prostate in rodents. In the rat ventral prostate, androgen depletion induces massive apoptosis and involution of the organ (Kyprianou et al, 1988). However, in an earlier study we found that only approximately one-third of the tumours from prostate cancer patients responded with an increase in apoptotic index 1 week after castration, and that this response was unrelated to the effects on proliferation (Westin et al, 1995a). The aim of this study was to investigate whether the short-term effects of castration therapy on morphology, apoptosis, proliferation and apoptosis-related oncogene expression in patients with prostate cancer are differential according to subsequent clinical response.

*Received 30 April 1997*
*Revised 7 July 1997*
*Accepted 11 July 1997*

*Correspondence to:* Pär Stattin, Department of Urology & Andrology, Umeå University, Umeå, S-901 85, Sweden

### MATERIAL AND METHODS

### Patients

At least three ultrasound-guided core biopsies were obtained using a spring-loaded 18 G BioptyCut gun shortly before and approximately a week after castration therapy in a series of patients with advanced prostate cancer. This procedure was approved by the local ethics committee, and the patients received information about the study and gave their consent. Local tumour stage was evaluated by rectal digital examination according to the UICC classification (1992). Metastatic status was evaluated by radionuclide bone scan at the time of diagnosis. To investigate the short-term effects in tumours from patients with distinctly different outcome, two groups of patients were selected. Response to castration therapy was defined as a serum level of PSA of $\leq 5$ ng ml$^{-1}$ 3 months after treatment, and non-response as a nadir PSA $\geq 10$ ng ml$^{-1}$. Using these definitions, 15 responding patients were selected; 2 out of 15 (13%) died of prostate cancer during the study period (mean observation time 19 months). A total of 13 non-responding patients were identified; 8 out of 13 (62%) died of prostate cancer during the study period. Mean cause-specific survival for PSA-responders was 37 months, and for the non-responders 19 months ($P = 0.004$); for patient characteristics see Table 1.

### Tumour tissue processing and evaluation

Core biopsies were fixed in Bouin's solution, embedded in paraffin, and cut in 4-μm-thick sections. Sections were stained

**Table 1** Clinical characteristics of patients prostate cancer treated with castration therapy

| | Responders ($n$ = 15)[a] | Non-responders ($n$ = 13) |
|---|---|---|
| Tumour stage[b] | | |
| T1–T2 | 6 | 1 |
| T3–T4 | 9 | 12 |
| Tumour grade[c] | | |
| G1, well differentiated | 4 | 2 |
| G2, intermediately | 7 | 4 |
| G3, poorly | 4 | 7 |
| Metastasis on bone scan | 10 | 10 |
| Patients age[d] at diagnosis (years) | 79 (74–84) | 76 (73–85) |
| PSA before therapy | 43 (16–123) | 396 (133–1635) |
| PSA nadir | 2 (1–4) | 76 (45–110) |
| Days between therapy and post-therapy biopsy | 7 (6–8) | 6 (4–7.5) |
| Number of patients dead because of disease | 2 | 8 |

[a]Response defined as PSA 5 ≤ ng ml$^{-1}$ 3 months after castration therapy, non-response defined as PSA ≥ 10 ng ml$^{-1}$ at 3 months after therapy. [b]Local tumour stage according to UICC 1992. [c]Tumour grade according to WHO (Mostofi et al, 1980). [d]Median values and 25% and 75% percentile values.

with haematoxylin and eosin. The tumour grade was evaluated according to the WHO classification system as: well, intermediately or poorly differentiated (Mostofi et al, 1980) (Table 1). All samples were analysed in a blind procedure by one observer without previous knowledge of the patients.

### Evaluation morphological response

The morphological response in the biopsies obtained after castration therapy was evaluated in terms of collapsed glandular acini and cytoplasmatic vacuolization (Dhom et al, 1982). The response was evaluated as all or none.

### Determination of apoptotic index

The apoptotic index (percentage of apoptotic cells) was determined by evaluating 2500 tumour cells per patient at 400 × magnification before and after castration therapy. Apoptotic cells were defined as single rounded cells or fragments with densely aggregated chromatin and condensed cytoplasm, often lying in 'halos' of extracellular space (Kerr et al, 1972). If more than one apoptotic body were seen per 'halo', these were considered to originate from the same cell and counted as one.

### Nuclear area

The nuclear area of 150 randomly selected tumour cells was measured and calculated using a MOP-Videoplan image analyser (Landström et al, 1990) (Kontron AG, Germany).

### Immunohistochemistry

Sections adjacent to the haematoxylin and eosin-stained sections were deparaffinized, rehydrated, incubated in 0.3% hydrogen peroxide in methanol, heated in a microwave oven for 5 × 4 min in

**Figure 1** Microphotograph of a prostate tumour before (**A**), and 7 days after (**B**) castration therapy, haematoxylin–eosin staining, 400 × magnification. Induction of cytoplasmatic vacuolization (open arrow) and glandular collapse (filled arrow) is apparent in the post-castration biopsy

0.01 M citrate buffer, pH 6, and incubated overnight with the following antibodies: anti-p53 (2 mg/l$^{-1}$, Ab 6, clone DO-1; Oncogene Science, Cambridge, MA, USA), anti-bax (2 mg/l$^{-1}$, bax P-19; Santa Cruz Biotechnology, Santa Cruz, CA, USA), anti-Fas (1 mg l$^{-1}$, Fas N-18; Santa Cruz Biotechnology), anti-Ki-67 (2 mg l$^{-1}$, MIB-1; Immunotech, Marseille, France) respectively. Sections were then incubated with a biotinylated secondary antibody, avidin–biotin complex reagents and peroxidase substrate for development. Between incubations, the sections were washed with phosphate-buffered saline. Bcl-2 immunoreactivity was obtained using the anti-bcl-2 antibody (0.67 mg l$^{-1}$, clone 124; Cambridge Biotechnology, Cambridge, MA, USA), and the supersensitive multilink kit (BioGenex, San Ramon, CA, USA), alkaline phosphatase and developed with fast red substrate.

Indexes (percentage of immunoreactive cells) for the apoptotic related oncoproteins p53 and bcl-2, and for the proliferation associated Ki-67 antigen (MIB-1) were determined by assessing the presence of immunoreactivity in at least 1000 randomly selected tumour cells at 400 × magnification with the aid of an eye-piece graticule in at least two slides from each tumour before and after

© Cancer Research Campaign 1998

**Table 2** Short-term effects after castration therapy in patients with advanced prostate cancer

| | Responders[a] | | Non-responders[b] | |
|---|---|---|---|---|
| | Before | After | Before | After |
| Proliferation index (%)[c] | 2.8 (1.3–5.1) | 1.1 (0.5–2)* | 5.3 (1.9–8.4) | 0.5 (0.1–2.6)** |
| Apoptotic index (%)[d] | 2.6 (1.0–3.7) | 3.5 (1.8–4.8)** | 2.8 (1.9–3.4) | 2.8 (1.9–3.8) |
| bcl-2 index (%) | 1.5 (0.4–8.5) | 10.0 (2.2–5.3) | 0.1 (0–3.8) | 2.7 (0.2–8.8)** |
| Nuclear area (m²)[e] | 24 (23–25) | 18 (17–21)** | 23 (22–25) | 19 (17–20)** |

Median values (25 and 75% percentiles). [a]Response defined as PSA 5 ≤ ng ml$^{-1}$ 3 months after castration therapy, non-response. [b]Non-response defined as PSA ≥ 10 ng ml$^{-1}$ at 3 months after therapy. [c]Proliferation measured by Ki-67 immunoreactivity with the MAb MIB-1. [d]Apoptotic signs evaluated on H and E-stained slides in 2500 cells. [e]Mean nuclear area calculated in 150 tumour cells morphometrically. *Difference before and after therapy ($P = 0.0534$). **Significant difference ($P < 0.05$) between before and after therapy.

**Table 3** Tumour response to castration therapy according to tumour grade

| Tumour grade[c] | Number of tumours | Number of tumours with | |
|---|---|---|---|
| | | Regressive morphology[a] (%) | Increased apoptotic index[b] (%) |
| G1 Well differentiated | 6 | 4 (66) | 4 (66) |
| G2 Intermediately | 11 | 8 (73) | 9 (82) |
| G3 Poor | 11 | 6 (55) | 6 (55) |

[a]Regressive morphology defined as collapsed glandular acini and cytoplasmic vacuolization (Dhom et al, 1982). [b]Apoptotic signs evaluated on H and E-stained slides in 2500 cells. [c]Tumour grade according to WHO (Mostofi et al, 1980). No significant trend for either regressive morphology or increased apoptotic index according to tumour grade by linear association test.

castration therapy. Immunoreactivity for bax and Fas was evaluated by subjectively estimating the number of reactive cells before and after therapy.

### Statistics

Non-parametric tests were applied to the results of the morphological and immunohistochemical investigations as these results did not appear to be normally distributed and the number of tumours was small. Difference for paired variables was tested by the Wilcoxon matched-pairs signed-ranks test, and with the Mann–Whitney $U$-test for unrelated samples. Fisher's exact test for independent samples was used for ordinal data. To test correlations between unrelated variables, the Spearman rank-sum test was applied. The median value was used for central tendency, and the 25% and 75% percentile limits were used as a measure of variability if not stated otherwise. Kaplan–Meier analysis was performed for survival, and the log-rank test was used to test the equality of the survival curves. In all tests, a two-tailed test of significance was applied, and a $P$-value less than 0.05 was considered significant (Norusis 1993).

### RESULTS

### Morphology

Castration induced morphological regressive changes in 18 out of 28 (64%) of the post-treatment biopsies (Figure 1). The magnitude of these changes varied among different tumours. In the group of responders, 14 out of 15 (93%) of the tumours showed regressive changes, compared with 4 out of 13 (31%) among the non-responders ($P < 0.001$). There was no significant correlation between the induction of morphological changes and tumour grade.

### Median nuclear area and cell proliferation rate (Ki-67 index)

The median tumour nuclear area was 23 μm² in the responding tumours, and 24 μm² in the non-responding group before therapy. After castration therapy, the median nuclear area decreased significantly to 18 μm² among responders, and to 19 μm² among non-responders, i.e. virtually the same decrease in the two groups. There was a wide range in the response in proliferation as measured by Ki-67 immunoreactivity. In a few tumours, Ki-67 index actually increased after therapy; however, the median Ki-67 index decreased in both groups. In the responding group, median Ki-67 index decreased from 2.8 to 1.1 ($P = 0.05$), whereas it decreased from 5.3 to 0.5 ($P = 0.04$) in the non-responding group (Table 2).

### Apoptotic index

The apoptotic index before and after therapy as well as the change in apoptotic rate was heterogeneous in both groups. The apoptotic index increased in 12 out of 15 (80%) of the responding tumours, and in 7 out of 13 (54%) of the non-responding tumours. In the responding groups, the median apoptotic index increased from 2.6 before to 3.5 after therapy ($P = 0.03$), whereas in the non-responding group, the median apoptotic index was unchanged at 2.8 before and

© Cancer Research Campaign 1998

after castration (Table 2). The median apoptotic index in the biopsies from patients that died of prostate cancer decreased non-significantly from 3.0 to 2.4, whereas it increased from 2.3 to 3.1 ($P$ = 0.008) in the 18 patients that did not succumb to the disease during the observation period. The indexes for apoptosis and proliferation measured before therapy correlated weakly with each other ($r_S$ = 0.48, $P$ = 0.009). Conversely, there were no significant correlations between the changes in apoptotic index and histological grade or morphological response. The distribution of tumours with regressive morphology and increased apoptotic index according to tumour grade is demonstrated in Table 3.

### Immunohistochemical detection of p53, bcl-2, bax, and Fas p53 immunoreactivity was present at low frequency in three tumours before castration therapy. The apoptotic index did not increase in these tumours. After therapy, three additional tumours were immunoreactive at a frequency of less than 5%, and these three tumours all belonged to the responding group. All p53-positive tumours were also positive for bcl-2, but no correlation between p53 and tumour differentiation, regressive morphology, nuclear area or Ki-67 index was seen.

Bcl-2 immunoreactivity was heterogeneous, parts of the tumours stained intensely whereas other parts did not stain at all. Bcl-2 index was low in most tumours, 21 out of 28 (75%) tumours had an index below 5% before castration. After castration therapy bcl-2 index increased in 19 out of 28 (68%) tumours, although it remained below 5% in 12 out of 28 (43%) tumours. Median bcl-2 index in the responding group increased from 1.5 to 10 after therapy ($P$ = 0.002, Table 2). In the non-responding tumours median bcl-2 index increased from 0.08 to 2.7 ($P$ = 0.05). There was a significant difference between responders and non-responders in the median bcl-2 index before therapy but not after therapy. There was no correlation between bcl-2 indexes and grade, morphological response or apoptotic index.

Bax immunoreactivity was present in the cytoplasm of virtually all epithelial and stromal cells in the tumours before and after therapy. In the benign epithelium, reactivity was most intense in the luminal cells. No increase in the number of reactive cells or intensity of bax immunoreactivity could be seen after castration therapy. No relation to histological grade, morphological response, apoptotic index or immunoreactivity for Ki-67, p53, bcl-2 or Fas was seen.

Fas immunoreactivity was also cytoplasmic and present in all tumours. Most epithelial tumour cells were stained, but stromal cells were negative. The number of reactive cells and intensity varied slightly between tumours. However, castration therapy did not affect the immunoreactivity for Fas. No correlation was found to grade, morphological response and apoptotic index. No immunoreactivity for Ki-67, p53, bcl-2 or bax was seen.

### DISCUSSION

### Regressive morphology

Tumour glandular and cellular morphology evaluated by light microscopy may be regarded as a measure of the sum of all genetic and epigenetic events in the cell, and tumour differentiation has repeatedly been shown to be a valid predictor for outcome in prostate tumour (Vesalainen et al, 1994). Most cellular effects of a change in the hormonal milieu in the normal prostate occur within days after the change (Kyprianou et al, 1988). However, little is known about the prognostic implications of the short-term morphological response to androgen deprivation in human prostate tumours. Collapse of glandular architecture and vacuolization of the cytoplasm were apparent in 64% of the tumours 1 week after androgen deprivation. Interestingly, a 68% long-term (6 months or more) morphological response rate was seen in tumours in patients treated with oestradiol (Dhom et al, 1982). The induction of regressive morphology was differential according to PSA response, indicating that the extent of early castration-induced cellular atrophy is related to PSA response and probably also to long-term outcome.

### Proliferation

Proliferation and cellular activity measured by nuclear area significantly decreased in almost all tumours, and similarly, a 90% decrease was observed in the mean serum PSA measured 3 months after therapy in both responders and non-responders. However, proliferation seemed dissociated from the tumour-suppressing effects leading to clinical response as the decrease was almost identical in the two groups. The reason for this somewhat surprising observation is unknown.

### Apoptosis

Human prostate tumours react with a decrease, no change or an increase in the apoptotic rate after androgen deprivation (Westin et al, 1995*a*). In this study, the median apoptotic index increased significantly after androgen deprivation in the group of PSA-responding tumours, whereas the median apoptotic index was unaffected in the non-responding group. To the best of our knowledge, this is the first study to show that the apoptotic response induced by castration in human prostate tumours is differential according to subsequent clinical outcome.

Together with our earlier results (Westin et al, 1995*a*), these data may reconcile contradictory data from experimental models; for instance, castration induced an increase in apoptotic rate, a decrease in proliferation rate and caused severe morphological destruction in PC-82 human prostate cancer grown in nude mice (Kyprianou et al, 1990; van Werden et al, 1993). Implants of the newly established LuCaP 23.1 tumour cell line grown in nude mice also underwent an increase in apoptosis and a decrease in proliferation after castration (Bladou et al, 1996). In contrast, androgen deprivation caused a decrease in both apoptotic rate and proliferation in the androgen-sensitive Dunning R3327 PAP grown in rat (Westin et al, 1995*b*), and the androgen sensitive Dunning G grown in mouse (Westin et al, 1997). Furthermore, no increase in apoptosis was seen after androgen deprivation in LnCaP xenografts (Gleave et al, 1992). Thus, these experimental models may each mimic aspects of the behaviour of human prostate tumours in individual patients after androgen deprivation.

### Bcl-2 and bax

Bcl-2 belongs to a family of oncoproteins regulating apoptosis, and has an anti-apoptotic function. Recent studies have shown that genetically engineered overexpression of the *bcl*-2 gene in androgen-sensitive prostate cancer cells confers resistance to androgen depletion in vivo (Raffo et al, 1995, Westin et al 1997). It has been speculated that *bcl*-2 expression would increase when a

© Cancer Research Campaign 1998

prostate tumour relapses. Two studies have shown bcl-2 immunoreactivity to be high in relapsed human prostate cancers (McDonnell et al, 1992; Colombel et al, 1993). However, median bcl-2 reactivity was significantly increased by androgen withdrawal in both groups in this series 1 week after therapy. Moreover, in non-relapsed human prostate cancers investigated at a mean time of 22 months after castration we observed that bcl-2 immunoreactivity was high (Stattin et al, 1996*b*). This indicates that an up-regulation of bcl-2 expression or a selection for bcl-2-expressing cells is permanently at work after androgen withdrawal in most human prostate tumours, and does not seem to be restricted to relapsed androgen-independent tumours. Moreover, recent studies have shown that bcl-2 can also exhibit antiproliferative effects (Pietenpol et al, 1994; Vairo et al, 1996). Thus, the down-regulation of the number of cycling cells seen in both groups in the present series may be related to the increase in bcl-2.

Another member of the bcl-2 family, the apoptosis-accelerating bax protein, was also studied. Bax immunoreactivity was widespread in all tumours, in accordance with an earlier study (Krajewska et al, 1996). Therefore, the balance between bcl-2 and bax immunoreactivity does not seem to explain the tendency of cells in prostate tumours to undergo apoptosis. Apparently, other proteins interacting with bcl-2 such as bcl-$x_l$, bcl-$x_s$, bag and bad will have to be explored to fully elucidate this issue.

### p53

Wild-type p53 is a tumour-suppressor gene regulating both proliferation and apoptosis, but the mutated form is a dominant oncogene (Greenblatt et al, 1994). The aberrant gene product of mutated p53 has a long half-life and can consequently be detected by immunoreactivity (Greenblatt et al, 1994). In the present series, p53 immunoreactivity was detected in 10% of the tumours before therapy, in accordance with the rate in most earlier series of prostate tumours [for a summary of the literature see (Stattin et al, 1996*a*)]. After castration, another three responding tumours were reactive at low frequency, this may be due to tumour heterogeneity and sampling of different cell populations or it may be due to an induction of wild-type p53. p53 immunoreactivity did not appear to be useful for predicting the outcome of castration therapy.

### Fas

Fas/APO-1/CD95 is a cell-surface receptor protein known to trigger apoptosis in a variety of cell types upon specific antibody binding, for example Fas ligand (Suzuki et al, 1996). In the normal mouse prostate, castration causes massive apoptosis, followed by a down-regulation of bcl-2 and induction of Fas expression. However, in Fas-lacking mice no involution of the prostate was seen after castration, suggesting a major role for Fas in castration-induced apoptosis in the prostate (Suzuki et al, 1996). Little is known about the expression of Fas in human prostate tumours. In this study, Fas immunoreactivity was present in all tumours, in line with what has been reported in 10 out of 11 tumour cell lines of human origin (Owen-Schaub et al, 1994). However, there was no difference in Fas expression between the responding and non-responding tumours, in accordance with what was described for Fas-positive cell lines (Owen-Schaub et al, 1994). This may suggest that defects in the Fas ligand or other factors downstream of Fas are crucial regulators of the Fas apoptotic pathway in some non-responding human prostate tumours.

### Conclusions

The results of this study suggest that some but not all of the short-term cellular effects of castration therapy in human prostate tumours are differential according to subsequent clinical response. Induction of glandular collapse and cytoplasmic vacuolization and an increase in apoptotic rate were related to favourable outcome. Conversely, the decrease in proliferative activity was similar in responding and non-responding tumours. Immunoreactivity for bcl-2, p53, bax and Fas was not differential according to response. This suggests that evaluation of signs of regressive morphology and apoptotic index on haematoxylin-stained sections in biopsies obtained before and shortly after castration therapy may be treatment-predictive for castration therapy. However, the results are based on a relatively small amount of tissue from a limited number of patients. There was a large range for nearly all the investigated parameters; this can at least partially be explained by tumour heterogeneity and the sampling of various tumour subpopulations with different characteristics at different time points. It is clear that a larger number of patients has to be studied, and that the number of biopsies obtained at each time point has to be increased to minimize these effects in future studies.

### ACKNOWLEDGEMENTS

Supported by grants from the Swedish Cancer Society (project no 1760), the Lions Cancer Research Foundation, Maud and Birger Gustavsson Foundation, Nilssons Foundation, and the University Hospital of Northern Sweden. We thank Elisabeth Dahlberg, Birgitta Ekblom and Pernilla Andersson for expert technical assistance.

### REFERENCES

Bladou F, Vessela RL, Buhler KR, Ellis WJ, True LD and Lange PH (1996) Cell proliferation and apoptosis during prostatic tumor xenograft involution and regrowth after castration. *Int J Cancer* **67**: 785–790

Bostwick DG, Burke HB, Wheeler TM, Chung LW, Bookstein R, Pretlow TG, Nagle RB, Montironi R, Lieber MM, Veltri RW, Grizzle WE and Grignon DJ (1994) The most promising surrogate endpoint biomarkers for screening candidate chemopreventive compounds for prostatic adenocarcinoma in short-term phase II clinical trials. *J Cell Biochem* **19** (suppl): 283–289

Colombel M, Symmans F, Gil S, O'Toole KM, Chopin D, Benson M, Olsson CA, Korsmeyer S and Buttyan R (1993) Detection of the apoptosis-suppressing oncoprotein bcl-2 in hormone-refractory human prostate cancers. *Am J Pathol* **143**: 390–400

Dhom G and Degro S (1982) Therapy of prostatic cancer and histopathologic follow-up. *Prostate* **3**: 531–542

Fowler Jr JE, Pandey P, Seaver LE, Feliz TP and Braswell NT (1995) Prostate specific antigen regression and progression after androgen deprivation for localized and metastatic prostate cancer. *J Urol* **153**: 1860–1865

Gleave ME, Hsieh J-T, Wu H-C, von Eschenbach AC and Chung LWK (1992) Serum prostate specific antigen levels in mice bearing human prostate LNCaP tumours are determined by tumor volume and endocrine and growth factors. *Cancer Res* **52**: 1598–1605

Greenblatt MS, Bennett WP, Hollstein M and Harris CC (1994) Mutations in the p53 tumor suppressor gene: clues to cancer etiology and molecular pathogenesis. *Cancer Res* **54**: 4855–4878

Kerr IF, Wyllie AH and Currie AR (1972) Apoptosis: a basic biological phenomenon with wide ranging implications in tissue kinetics. *Br J Cancer* **26**: 239–257

Krajewska M, Krajewski S, Epstein JI, Shabaik A, Sauvageot J, Song K, Kitada S and Reed JC (1996) Immunohistochemical analysis of bcl-2, bax, bcl-X, and mcl-1 expression in prostate cancers. *Am J Pathol* **148**: 1567–1576

Kyprianou N and Isaacs JT (1988) Activation of programmed cell death in the rat ventral prostate after castration. *Endocrinology* **122**: 552–562

© Cancer Research Campaign 1998

Kyprianou N, English HF and Isaacs JT (1990) Programmed cell death during regression of PC-82 human prostate cancer following androgen ablation. *Cancer Res* **50**: 3748–3753

Landström M, Bergh A, Tomic R and Damber JE (1990) Estrogen treatment combined with castration inhibits tumor more effectively than castration alone in the Dunning R-3327 rat prostatic adenocarcinoma. *Prostate* **17**: 57–68

McDonnell TJ, Troncoso P, Brisbay SM, Logothetis C, Chung LWK, Hsieh JT, Tu SM and Campbell ML (1992) Expression of the protooncogene bcl-2 in the prostate and its association with emergence of androgen-independent prostate cancer. *Cancer Res* **52**: 6940–6944

Mostofi FK, Sesterhenn IA and Sobin LH (1980) *International Histological Classification of Prostate Tumours*. WHO: Geneva

Norusis MJ (1993) *SPSS for Windows*. SPSS: Chicago

Owen-Schaub LB, Radinsky R, Kruzel E, Berry K and Yonehara S (1994) Anti-Fas on nonhematopoetic tumors: levels of Fas/APO-1 and bcl-2 are not predictive of biological responsiveness. *Cancer Res* **54**: 1580–1586

Petros JA and Andriole GL (1993) Serum PSA after antiandrogen therapy. *Urol Clin N Am* **20**: 749–756

Pietenpol JA, Papadopoulos N, Markowitz S, Wilson JKV, Kinzler KW and Vogelstein B (1994) Paradoxical inhibition of solid tumor cell growth by bcl-2. *Cancer Res* **54**: 3714–3717

Raffo AJ, Perlman H, Chen MW, Day ML, Streitman JS and Buttyan R (1995) Overexpression of bcl-2 protects prostate cancer cells from apoptosis in vitro and confers resistance to androgen depletion in vivo. *Cancer Res* **55**: 4438–4445

Resnick MI and Grayhack JT (1975) Treatment of stage IV carcinoma of the prostate. *Urol Clin N Am* **2**: 141–161

Stattin P, Bergh A, Karlberg L, Nordgren H and Damber JE (1996*a*) p53 immunoreactivity as prognostic marker for cancer specific survival in prostate cancer. *Eur Urol* **30**: 65–72

Stattin P, Damber JE, Karlberg L, Nordgren H and Bergh A (1996*b*) Bcl-2 immunoreactivity in prostate tumorigenesis in relation to prostatic intraepithelial neoplasia. Grade, hormonal status, metastatic growth and survival. *Urol Res* **24**: 257–264

Suzuki A, Matsuzawa A and Iguchi T (1996) Down regulation of Bcl-2 is the first step on Fas-mediated apoptosis of male reproductive tract. *Oncogene* **13**: 31–37

UICC (1992) *TNM Classification of Malignant Tumours*, 4th edn. Hermanek P and Sobin LH (eds). Springer: Berlin

Vairo G, Innes KM and Adams JM (1996) Bcl-2 has a cell cycle inhibitory function separable from its enhancement of cell survival. *Oncogene* **13**: 1511–1519

van Werden WM, van Kreuningen A, Elissen NMJ, de Veermeji M, Jong FH, van Steenbrugge GJ and Schröder FH (1993) Castration-induced changes in morphology, androgen levels, and proliferative activity of human prostate cancer tissue grown in athymic mice. *Prostate* **23**: 149–164

Vesalainen S, Lipponen P, Talja M and Syrjänen K (1994) Histological grade, Perineural infiltration, tumour-infiltrating lymphocytes and apoptosis as determinants of long-term prognosis in prostatic adenocarcinoma. *Eur J Cancer* **30A**: 1797–1803

Westin P, Stattin P, Damber JE and Bergh A (1995*a*) Castration therapy rapidly induces apoptosis in a minority and decreases cell proliferation in a majority of human prostatic tumors. *Am J Pathol* **146**: 1368–1375

Westin P, Brändström A, Damber JE and Bergh A (1995*b*) Castration plus oestrogen treatment induces but castration alone suppresses epithelial cell apoptosis in an androgen-sensitive prostatic adenocarcinoma. *Br J Cancer* **72**: 140–145

Westin P, Lo P, Marin MC, Fernandes A, Sarkiss M and McDonnell TJ (1997) Bcl-2 expression confers androgen independence in an androgen sensitive prostatic carcinoma *in vivo*. *Int J Oncol* in press

© Cancer Research Campaign 1998